# Supplementary material for: Ultrafast one-minute electronic detection of SARS-CoV-2 infection by 3CLpro enzymatic activity in untreated saliva samples
Source: Nat Commun. 2022 Oct 26;13:6375. doi: 10.1038/s41467-022-34074-2 (PMC9605950; doi:10.1038/s41467-022-34074-2)
Supplement: Supplementary file 1 — Supplementary Information [file 41467_2022_34074_MOESM1_ESM.pdf]

Supporting information

UltraFast One-Minute Electronic Detection of SARS-CoV-2 Infection by 3CL^{pro} Enzymatic Activity in Untreated Saliva Samples

Ella Borberg[1], Eran Granot[1], and Fernando Patolsky[1,2]

1. School of Chemistry, Faculty of Exact Sciences, Tel Aviv University, Tel Aviv 69978, Israel.
2. Department of Materials Science and Engineering, the Iby and Aladar Fleischman Faculty of Engineering, Tel Aviv University, Tel Aviv 69978, Israel.

Emails: fernando@post.tau.ac.il

Table of Contents

Supplementary Table 1 3
Supplementary Fig.1 4
Supplementary Fig.2 4
Supplementary Fig.3 5
Supplementary Fig.4 5
Supplementary Fig.5 6
Supplementary Fig.6 6
Supplementary Fig.7 7
Supplementary Fig.8 7
Supplementary Discussion 8
Supplementary References 9

Supplementary Table 1 | Protein information and sequences.

Construct name	Composition	Sequence	Mw (kDa)
SARS-CoV-2 3CL^{pro} Enzyme	Recombinant, Escherichia coli-derived	SGFR KMAF PSGK VEGC MVQV TCGT TTLN GLWL DDVV YCPR HVIC<br>TSED MLNP NYED LLIR KSNH NFLV QAGN VQLR VIGH SMQN CVLK<br>LKVD TANP KTPK YKFV RIQP GQTF SVLA CYNG SPSG VYQC AMRP<br>NFTI KGSF LNGS CGSV GFNI DYDC VSFC YMHH MELP TGVH AGTD<br>LEGN FYGP FVDR QTAQ AAGT DTTI TVNV LAWL YAAV INGD RWFL<br>NRFT TTLN DFNL VAMK YNYE PLTQ DHVD ILGP LSAQ TGIA VLDM<br>CASL KELL QNGM NGRT ILGS ALLE DEFT PFDV VRQC SGVT FQ	34
SARS-CoV-2 3CL^{pro} Substrate	Peptide	KTSA VLQS GFRK ME	1.6
SARS-CoV-2 3CL^{pro} Antibody	Polyclonal, Rabbit-Derived	Target:<br>SMQN CVLK LKVD TANP KTPK YKFV RIQP GQTF SVLA CYNG SPSG<br>VYQC AM	~150
BSA	Native Bovine Serum Albumin Protein	MKWV TFIS LLLL FSSA YSRG VFRR DTHK SEIA HRFK DLGE EHFK<br>GLVL IAFS QYLQ QCPF DEHV KLVN ELTE FAKT CVAD ESHA GCEK<br>SLHT LFGD ELCK VASL RETY GDMA DCCE KQEP ERNE CFLS HKDD<br>SPDL PKLK PDPN TLCD EFKA DEKK FWGK YLYE IARR HPYF YAPE<br>LLYY ANKY NGVF QECC QAED KGAC LLPK IETM REKV LASS ARQR<br>LRCA SIQK FGER ALKA WSVA RLSQ KFPK AEFV EVTK LVTD LTKV<br>HKEC CHGD LLEC ADDR ADLA KYIC DNQD TISS KLKE CCDK PLLE<br>KSHC IAEV EKDA IPEN LPPL TADF AEDK DVCK NYQE AKDA FLGS<br>FLYE YSRR HPEY AVSV LLRL AKEY EATL EECC AKDD PHAC YSTV<br>FDKL KHLV DEPQ NLIK QNCD QFEK LGEY GFQN ALIV RYTR KVPQ<br>VSTP TLVE VSRS LGKV GTRC CTKP ESER MPCT EDYL SLIL NRLC<br>VLHE KTPV SEKV TKCC TESL VNRR PCFS ALTP DETY VPKA FDEK<br>LFTF HADI CTLP DTEK QIKK QTAL VELL KHKP KATE EQLK TVME<br>NFVA FVDK CCAA DDKE ACFA VEGP KLVV STQT ALA	69
Myoglobin Antibody	Monoclonal, Rabbit-derived	Target:<br>GHHE AEIK PLAQ SHAT KHKI PVKY LEFI SECI IQVL QSKH PGDF<br>GADA QGAM NKAL ELFR KDMA SNYK ELGF QG	~150
GFP	Recombinant, Escherichia coli-derived	MSKG EELF TGVV PILV ELDG DVNG HKFS VSGE GEGD ATYG KLTL<br>KFIC TTGK LPVP WPTL VTTF SYGV QCFS RYPD HMKQ HDFF KSAM<br>PEGY VQER TIFF KDDG NYKT RAEV KFEG DTLV NRIE LKGI DFKE<br>DGNI LGHK LEYN YNSH NVYI MADK QKNG IKVN FKIR HNIE DGSV<br>QLAD HYQQ NTPI GDGP VLLP DNHY LSTQ SALS KDPN EKRD HMVL<br>LEFV TAAG ITHG MDEL YK	27
Human TMPRSS2	Recombinant, Wheat germ-derived	GWGA TEEK GKTS EVLN AAKV LLIE TQRC NSRY VYDN LITP AMIC<br>AGFL QGNV DSCQ GDSG GPLV TSKN NIWW LIGD TSWG SGCA KAYR<br>PGVY GNVM VFTD WIYR QMRA DG	38
MERS-CoV 3CL Protease	Recombinant, Escherichia coli-derived	SGLV KMSH PSGD VEAC MVQV TCGS MTLN GLWL DNTV WCPR HVMC<br>PADQ LSDP NYDA LLIS MTNH SFSV QKHI GAPA NLRV VGHA MQGT<br>LLKL TVDV ANPS TPAY TFTT VKPG AAFS VLAC YNGR PTGT FTVV<br>MRPN YTIK GSFL CGSC GSVG YTKE GSVI NFCY MHQM ELAN GTHT<br>GSAF DGTM YGAF MDKQ VHQV QLTD KYCS VNVV AWLY AAIL NGCA<br>WFVK PNRT SVVS FNEW ALAN QFTE FVGT QSVD MLAV KTGV AIEQ<br>LLYA IQQL YTGF QGKQ ILGS TMLE DEFT PEDV NMQI MGVV MQ	34
SARS-CoV 3CL Protease	Recombinant, Escherichia coli-derived	SGFR KMAF PSGK VEGC MVQV TCGT TTLN GLWL DDTV YCPR HVIC<br>TAED MLNP NYED LLIR KSNH SFLV QAGN VQLR VIGH SMQN CLLR<br>LKVD TSNP KTPK YKFV RIQP GQTF SVLA CYNG SPSG VYQC AMRP<br>NHTI KGSF LNGS CGSV GFNI DYDC VSFC YMHH MELP TGVH AGTD<br>LEGK FYGP FVDR QTAQ AAGT DTTI TLNV LAWL YAAV INGD RWFL<br>NRFT TTLN DFNL VAMK YNYE PLTQ DHVD ILGP LSAQ TGIA VLDM<br>CAAL KELL QNGM NGRT ILGS TILE DEFT PFDV VRQC SGVT FQ	34
HIV-2 Protease	Recombinant, Escherichia coli-derived	PQFS LWKR PVVT AHIE GQPV EVLL DTGA DDSI VAGI ELGS NYSP<br>KIVG GIGG FINT KEYK NVEI EVLN KRVR ATIM TGDT PINI FGRN<br>ILAS LGMS LNL	11
CA-15.3	Recombinant, Escherichia coli-derived	LRPG SVVV QLTL AFRE GTIN VHDV ETQF NQYK TEAA SRYN LTIS<br>DVSG	14

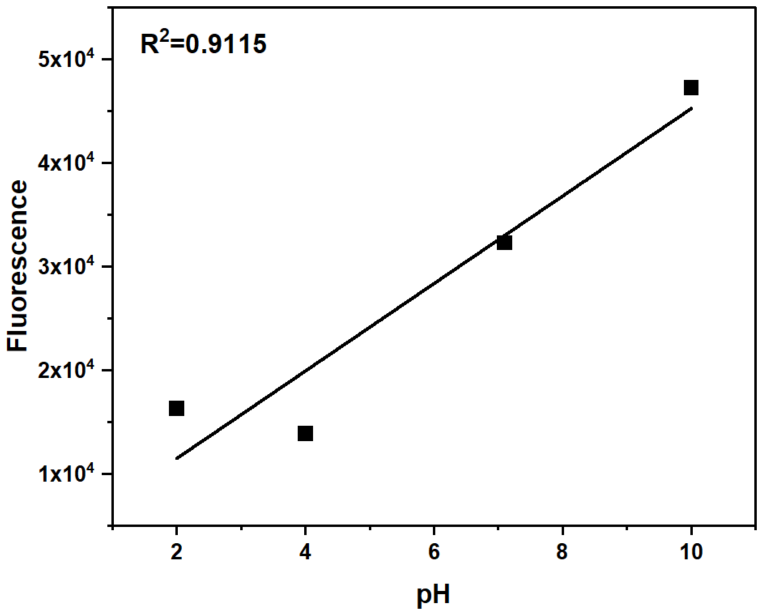

Supplementary Fig.1 | Calibration curve of 8-Hydroxypyrene-1,3,6-trisulfonic acid fluorescence as a function of pH.

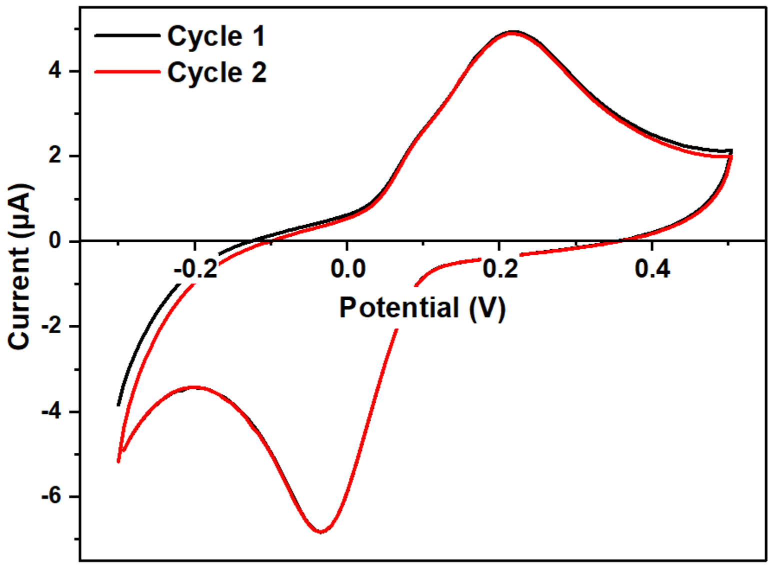

Supplementary Fig.2 | Repetitiveness of CV cycles. 2 cycles of CV measurements of pBQ (15 μM) in PB (900 μl, 25 mM), NaCl (75mM), pH 7.65. Scan rate: 0.1 V sec^{-1}, vs. Ag/AgCl.

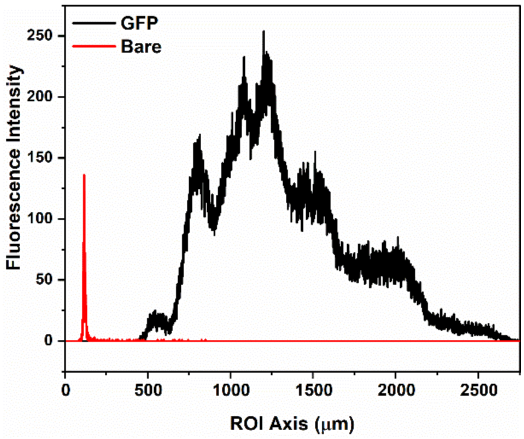

Supplementary Fig.3 | Protein permeability through CPE. GFP fluorescence intensity on bare (red) and GFP-modified carbo paper. Correlating to **Figure 3a and b**.

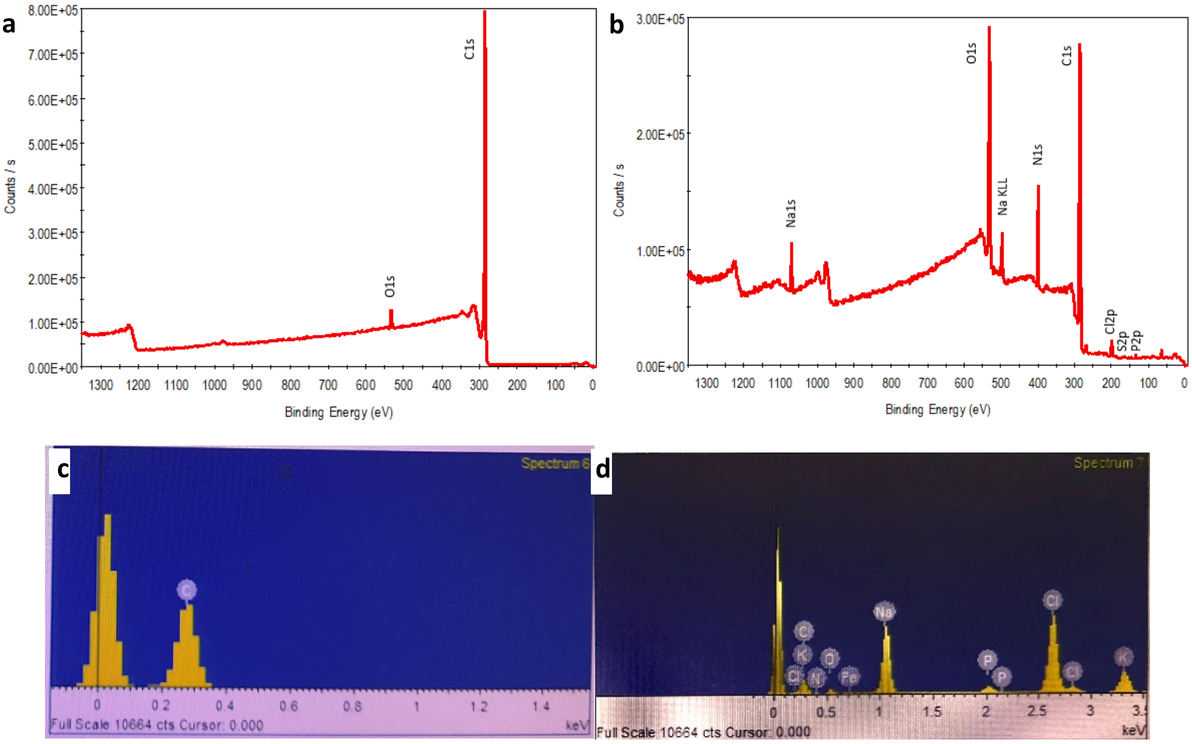

Supplementary Fig.4 | X-Ray Photoelectron Spectroscopy representative survey spectra for bare CPE (a) and antibody treated CPE (b). Energy-dispersive X-ray spectroscopy representative survey spectra for bare CPE (c) and antibody treated CPE (d). Correlating to **Figure 3g and h**. Atomic percentage was calculated in ThermoScientific *Avatage* software, using the following equation:

$$C_A = \frac{I_A/S_A}{\sum_n I_n/S_n} \times 100\%$$

Where C_A is the atomic % content of A, I_A is the intensity of an atoms' peak and S_A is the sensitivity of the atom.

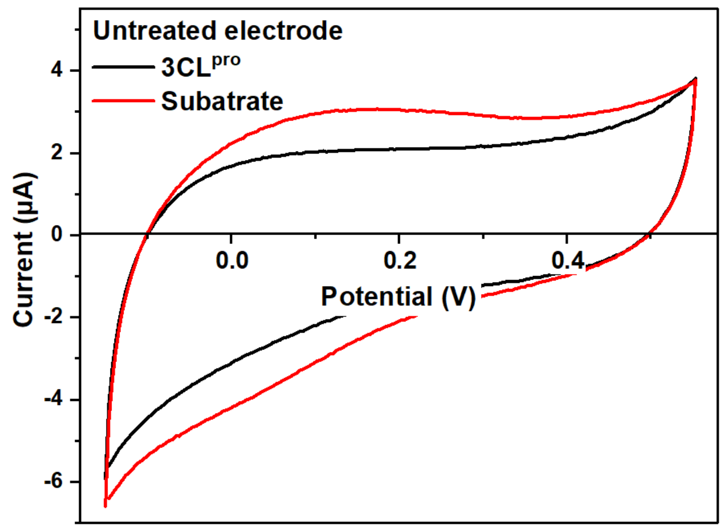

Supplementary Fig.5 | Evidence of RedOx pH indicator necessity. CV of untreated CPE obtained with 3CL^{pro} (black curve) and 3CL^{pro}-substrate (red curve) in the absence of pBQ. 900 µl of 80 nM 3CL^{pro}, 25 µM PB, 75mM NaCl, pH 7.4, scan rate 0.1 V sec^{-1}, vs. Ag/AgCl.

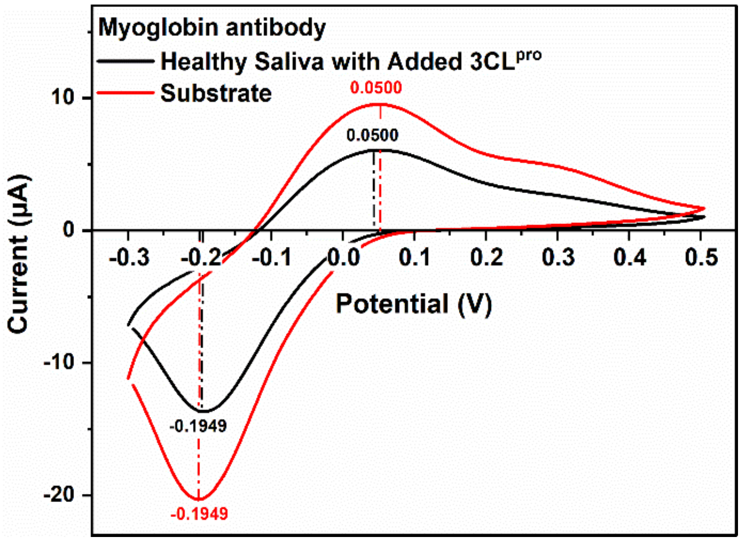

Supplementary Fig.6 | Antibody specificity. CV curves of CPE treated with myoglobin antibody and exposed to SARS-CoV-2 negative saliva spiked with 0.2 pmol 3CL^{pro} before (black) and after (red) exposure to 3CL^{pro} substrate. CV curves were obtained in 900 µl of 15 µM pBQ, 25 µM PB, 75mM NaCl, pH 7.4, scan rate 0.1 V sec^{-1}, vs. Ag/AgCl.

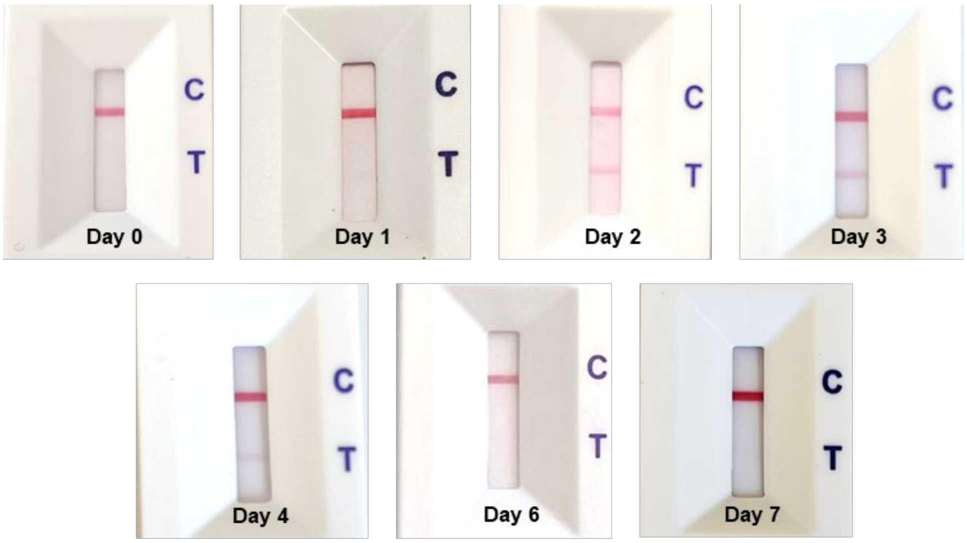

Supplementary Fig.7 | Images of COVID-19 Antigen Rapid Test results over infection period, correlating to **Figure 5e**.

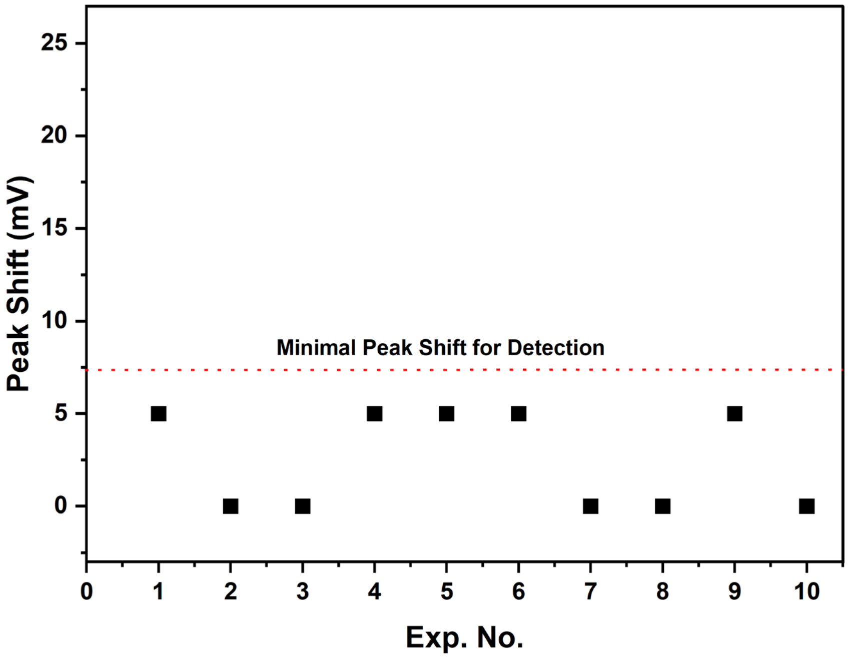

Supplementary Fig.8 | pBQ oxidation peak shift results of 10 consecutive experiments measuring the same healthy saliva sample.

Supplementary Discussion

$3CL^{pro}$ pH change derived calculation

Measurements of $3CL^{pro}$ activity showed 80 pmol $3CL^{pro}$ activity in the presence of 8 nmol $3CL^{pro}$ substrate results in a pH drop of 0.63 units (starting pH was 7.51 and final pH was 6.88) in 2 minutes, see **Figure 2A**. Since $3CL^{pro}$ tends to dimerize,[1] and the turnover rate of our $3CL^{pro}$ substrate is about 60 substrate molecules per minute,[2] the activity of $3CL^{pro}$ could be calculated by predicting that 80 pmol $3CL^{pro}$ would cleave 1.2-2.4 nmol of the substrate in 120 seconds, for 80 μl well:

$$\text{KTSAVLQSGFRKME} \xrightarrow{3CL^{pro}} \text{KTSAVLQ(COOH)} + (\text{H}_2\text{N})\text{SGFRKME}$$

$$\text{KTSAVLQ(COOH)} \rightleftharpoons \text{KTSAVLQ(COO}^-) + H^+$$

$$\frac{d[H^+]}{dt} = k[\text{KTSAVLQ(COOH)}] \rightarrow \frac{0.101\ \mu M}{120\ sec} = k \times (1.5 - 3.0\ \mu M) \rightarrow$$

$$k = (2.8 - 5.6)x10^{-4}\frac{1}{sec}$$

For 900 μl cell with the same presence of $3CL^{pro}$, and starting pH is 7.40:

$$\frac{d[H^+]}{dt} = (2.8 - 5.6)x10^{-4}\frac{1}{sec} \times (0.13 - 0.27\ \mu M) \rightarrow\ d[H^+] = 0.05 - 0.18\ \mu M$$

$$[H^+]_f = 0.09 - 0.22\ \mu M$$

$$\rightarrow pH = 6.66 - 7.05 \rightarrow\ \Delta pH = 0.35 - 0.74$$

Supplementary References

1. El-Baba, T. J. *et al.* Allosteric Inhibition of the SARS-CoV-2 Main Protease: Insights from Mass Spectrometry Based Assays**. *Angewandte Chemie International Edition* **59**, 23544–23548 (2020).
2. Kao, R. Y. *et al.* Characterization of SARS-CoV main protease and identification of biologically active small molecule inhibitors using a continuous fluorescence-based assay. *FEBS Letters* **576**, 325–330 (2004).
