# Supplementary material for: Ultrafast one-minute electronic detection of SARS-CoV-2 infection by 3CLpro enzymatic activity in untreated saliva samples
Source: Nat Commun. 2022 Oct 26;13:6375. doi: 10.1038/s41467-022-34074-2 (PMC9605950; doi:10.1038/s41467-022-34074-2)
Supplement: Supplementary file 3 — Reporting Summary [file 41467_2022_34074_MOESM3_ESM.pdf]

nature portfolio

Corresponding author(s): Fernando Patolsky

Last updated by author(s): Oct 11, 2022

Reporting Summary

Nature Portfolio wishes to improve the reproducibility of the work that we publish. This form provides structure for consistency and transparency in reporting. For further information on Nature Portfolio policies, see our Editorial Policies and the Editorial Policy Checklist.

Statistics

For all statistical analyses, confirm that the following items are present in the figure legend, table legend, main text, or Methods section.

n/a	Confirmed	
☐	☒	The exact sample size (n) for each experimental group/condition, given as a discrete number and unit of measurement
☐	☒	A statement on whether measurements were taken from distinct samples or whether the same sample was measured repeatedly
☒	☐	The statistical test(s) used AND whether they are one- or two-sided<br>*Only common tests should be described solely by name; describe more complex techniques in the Methods section.*
☒	☐	A description of all covariates tested
☒	☐	A description of any assumptions or corrections, such as tests of normality and adjustment for multiple comparisons
☐	☒	A full description of the statistical parameters including central tendency (e.g. means) or other basic estimates (e.g. regression coefficient) AND variation (e.g. standard deviation) or associated estimates of uncertainty (e.g. confidence intervals)
☒	☐	For null hypothesis testing, the test statistic (e.g. F, t, r) with confidence intervals, effect sizes, degrees of freedom and P value noted<br>*Give P values as exact values whenever suitable.*
☒	☐	For Bayesian analysis, information on the choice of priors and Markov chain Monte Carlo settings
☒	☐	For hierarchical and complex designs, identification of the appropriate level for tests and full reporting of outcomes
☒	☐	Estimates of effect sizes (e.g. Cohen's d, Pearson's r), indicating how they were calculated

Our web collection on statistics for biologists contains articles on many of the points above.

Software and code

Policy information about availability of computer code

Data collection	PSTrace software 5.8. i-control software 2.0.
Data analysis	Origin 2020. Microsoft Excel 2016.

For manuscripts utilizing custom algorithms or software that are central to the research but not yet described in published literature, software must be made available to editors and reviewers. We strongly encourage code deposition in a community repository (e.g. GitHub). See the Nature Portfolio guidelines for submitting code & software for further information.

Data

Policy information about availability of data

All manuscripts must include a data availability statement. This statement should provide the following information, where applicable:

- Accession codes, unique identifiers, or web links for publicly available datasets
- A description of any restrictions on data availability
- For clinical datasets or third party data, please ensure that the statement adheres to our policy

All data generated or analysed during this study are included in this published article (and its supplementary information files).

Field-specific reporting

March 2021

Life sciences study design

All studies must disclose on these points even when the disclosure is negative.

Sample size	Sample size for each clinical group, healthy versus infected, is provided in paper. Sample size was determined based on donors availability and in order to acquire a statistically meaningful minimum number of donors for each group in order to demonstrate the effectivity of the developed diagnostic method. No pre-calculations on the sample size were performed.
Data exclusions	No data was excluded from the paper. All clinical samples performed and their respective results were completely included and no single experiment was excluded.
Replication	Each sample test in the manuscript was repeated at least twice, five times in selected cases, to demonstrate the accuracy of the detection approach. All attempts at replication were successful.
Randomization	Healthy individual samples were validated by PCR-negative COVID 19 results, while COVID infected individuals cohort were validated by PCR test as well. Individuals priorly tested positive for Covid19 were enrolled as donors for the performance of our test. Individuals tested negative by PCR for Covid19 were enrolled as healthy donors. No randomization was applied.
Blinding	The nature of the samples tested, either infected or healthy, was unknown to the researchers of this study during the tests. After receiving the results of this approach, the results of the presented platform were compared to the PCR results for all the tested individuals.

Reporting for specific materials, systems and methods

We require information from authors about some types of materials, experimental systems and methods used in many studies. Here, indicate whether each material, system or method listed is relevant to your study. If you are not sure if a list item applies to your research, read the appropriate section before selecting a response.

Materials & experimental systems

n/a	Involved in the study	
☐	☒	Antibodies
☒	☐	Eukaryotic cell lines
☒	☐	Palaeontology and archaeology
☒	☐	Animals and other organisms
☐	☒	Human research participants
☒	☐	Clinical data
☒	☐	Dual use research of concern

Methods

n/a	Involved in the study	
☒	☐	ChIP-seq
☒	☐	Flow cytometry
☒	☐	MRI-based neuroimaging

Antibodies

Antibodies used	3CLpro enzyme (Recombinant derived from Escherichia coli, ab277614, ABCAM), 3CLpro specific antibody (Rabbit-derived polyclonal, NBP3-07062, Novous biological), 3CLpro substrate (peptide, KTSAVLQSGFRKME, Sigma-Aldrich), GFP (Recombinant derived from Escherichia coli, ab84191, ABCAM), Myoglobin antibody (Monoclonal rabbit-derived, ab77232, ABCAM), HIV-2 Protease (Recombinant derived from Escherichia coli, ab84117, ABCAM), CA-15.3 (Recombinant derived from Escherichia coli, ab80082, ABCAM), Human TMPRSS2 protein (Recombinant derived from Wheat germ, ab112364, ABCAM), MERS-CoV 3CL Protease (Recombinant derived from Escherichia coli, E-719, Novous biological), SARS-CoV 3CL Protease (Recombinant derived from Escherichia coli, E-718, Novous biological), Chymotrypsin protein (Native human, ab90927, ABCAM).<br>The immunogen is located within 80-130 amino acids of the SARS-CoV-2 (COVID-19) 3CL-PRO (NSP5) protein. Supplied by Novus Biologicals LTD
Validation	3CLpro specific antibody: raised against a peptide corresponding to 15 amino acids in the center of SARS-CoV-2 3CLpro protein. The immunogen is located within 80-130 amino acids of the SARS-CoV-2 3CL-PRO. Western Blot Validation with SARS-CoV-2 3CL Protease Protein. Loading: 30 ng per lane of SARS-CoV-2 3CL Protease recombinant protein.<br>Myoglobin antibody: Western Blot Validation with Myoglobin antibody at 1/5000 dilution.

Human research participants

Policy information about studies involving human research participants

Population characteristics	The human participants were female and male adults from the age of 18 years old up to the year of 78 years old. No additional clinical information on donors was collected during the study.
Recruitment	COVID 19 infected participants were recruited after a PCR-positive test. Healthy individuals were recruited and validated as COVID 19-negative after PCR test. No self-selection bias or other biases has occurred during the whole study that may have affected the results presented. An informed consent was obtained from participants and participants were not compensated.
Ethics oversight	Tel Aviv University Ethics Committee (IRB protocol number 71.19)

Note that full information on the approval of the study protocol must also be provided in the manuscript.
